# Mechanically Robust Hydrogels Facilitating Bone Regeneration through Epigenetic Modulation

**DOI:** 10.1002/advs.202203734

**Published:** 2022-09-25

**Authors:** Tingting Yu, Lingyun Zhang, Xueyu Dou, Rushui Bai, Hufei Wang, Jie Deng, Yunfan Zhang, Qiannan Sun, Qian Li, Xing Wang, Bing Han

**Affiliations:** ^1^ Department of Orthodontics Peking University School and Hospital of Stomatology Beijing 100081 China; ^2^ National Center of Stomatology & National Clinical Research Center for Oral Diseases & National Engineering Laboratory for Digital and Material Technology of Stomatology & Beijing Key Laboratory for Digital Stomatology & Research Center of Engineering and Technology for Computerized Dentistry Ministry of Health & NMPA Key Laboratory for Dental Materials Beijing 100081 China; ^3^ Beijing National Laboratory for Molecular Sciences Institute of Chemistry Chinese Academy of Sciences Beijing 100190 China; ^4^ University of Chinese Academy of Sciences Beijing 100049 China

**Keywords:** bone tissue engineering, epigenetic regulation, hydrogels, mesenchymal stem cells, polyhedral oligomeric silsesquioxane

## Abstract

Development of artificial biomaterials by mimicking extracellular matrix of bone tissue is a promising strategy for bone regeneration. Hydrogel has emerged as a type of viable substitute, but its inhomogeneous networks and weak mechanics greatly impede clinical applications. Here, a dual crosslinked gelling system is developed with tunable architectures and mechanics to promote osteogenic capacity. Polyhedral oligomeric silsesquioxane (POSS) is designated as a rigid core surrounded by six disulfide‐linked PEG shells and two 2‐ureido‐4[1H]‐pyrimidinone (UPy) groups. Thiol‐disulfide exchange is employed to fabricate chemical network because of the pH‐responsive “on/off” function. While self‐complementary UPy motif is capable of optimizing local microstructure to enhance mechanical properties. Taking the merits of biocompatibility and high‐mechanics in periodontal ligament stem cells (PDLSCs) proliferation, attachment, and osteogenesis, hybrid hydrogel exhibits outstanding osteogenic potential both in vitro and in vivo. Importantly, it is the first time that a key epigenetic regulator of ten‐eleven translocation 2 (Tet2) is discovered to significantly elevate the continuously active the WNT/*β*‐catenin through Tet2/HDAC1/E‐cadherin/*β*‐catenin signaling cascade, thereby promoting PDLSCs osteogenesis. This work represents a general strategy to design the hydrogels with customized networks and biomimetic mechanics, and illustrates underlying osteogenic mechanisms that will extend the design rationales for high‐functional biomaterials in tissue engineering.

## Graphical abstract

A dual‐crosslinked gelling system with tunable architectures and mechanical properties can promote periodontal ligament stem cell‐mediated bone regeneration by activating WNT/*β*‐catenin through translocation 2 epigenetic regulation.

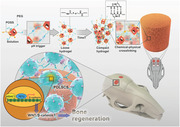

## 1 Introduction

Craniofacial bone defects resulting from trauma, infection, or tumor resection can result in loss of maxillofacial function and present numerous social, psychological, and emotional challenges, which impact patient quality of life significantly. Current clinical strategies for bone tissue transplantation typically involve the use of autogenous, allogeneic, and/or prosthetic materials, which are used in more than 90% of graft surgeries.^[^ ^1^ ^]^ However, the invasive surgery process, lack of tissue donors, and high cost are severe drawbacks to bone tissue transplantation.^[^ ^1b^ ^]^

Hydrogels have been extensively explored in the tissue engineering field owing to their good biocompatibility, biodegradation, and tunable physicochemical properties for bone regeneration. However, their inhomogeneous networks and weak mechanical properties greatly impede their clinical application for hard tissue regeneration. Current methodologies for tailoring hydrogels typically involve manipulating multiple external stimuli, such as pH, temperature, redox, and light.^[^ ^2^ ^]^ These methods, however, are prone to rely on the preset crosslinker content and involve the issues of non‐immediate in situ regulation and tedious operations, making it difficult to precisely control the cross‐linking evolution once the gel formation is initiated. In addition, these chemical crosslinked hydrogels are extremely brittle and fragile due to the compact accumulation of hydrophobic cores and insufficient flexible chains for energy dissipation.^[^ ^3^ ^]^ To avoid these problems, major efforts have been devoted to optimizing the cross‐linking process to increase mechanical performances by implementing energy dissipation mechanisms and distinctive structures via the formation of multiple‐network.^[^ ^4^ ^]^ For example, the hydrogels can be directly incorporated with the reinforcing nanoparticles (e. g. silicates, graphene, calcium phosphate) or durable networks as the main constituent of noncovalent interactions via physical blending or chemical attachment. Under this circumstance, hierarchical hydrogen‐bonding interactions can endow the composite hydrogels with excellent self‐adaptive capacity and high energy dissipation upon exposure to external stress.

Polyhedral oligomeric silsesquioxane (POSS) nanocages offer a striking alternative as it possesses a rigid cage‐like core surrounded by eight organic corner groups that can be functionalized for modification.^[^ ^5^ ^]^ Thanks to its excellent thermal and mechanical stabilities as well as simplicity in processing, POSS has been an important building block to generating a myriad of POSS‐incorporated hydrogels, which present a homogeneous system for developing high‐performance materials in diverse applications.^[^ ^6^ ^]^ However, these hydrogels are extremely brittle and fragile because of the compact accumulation of hydrophobic cores and insufficient flexible chains for energy dissipation, which relatively hampered their potential applications. Therefore, it is a prerequisite to have reversible interactions to construct strong and tough materials to maintain structural stability when such hybrid hydrogels are exposed to external stress. In views of the biomimicking modular design of skeletal muscle protein titin that possesses a remarkable combination of strength, toughness, and elasticity by reversible rupture of intramolecular secondary interactions, quadruple hydrogen bonding 2‐ureido‐4[1H]‐pyrimidinone (UPy) motifs have been widely pioneered and developed as an effective noncovalent interaction in the construction of self‐adaptive materials.^[^ ^7^ ^]^ Hence, UPy is the potential candidate to fabricate toughened hydrogels with controlled properties. Interestingly, incorporation of UPy motifs to enhance the self‐healing POSS‐based materials is only an emerging field in aerospace coating materials,^[^ ^8^ ^]^ and their application in bone repair and regeneration has to the best of our knowledge not been proposed, especially in repairing connective tissue bone defects in vivo.

In recent years, POSS units possessed excellent osteogenic effects,^[^ ^9^ ^]^ but the osteogenic mechanism is relatively obscure, which is simply ascribed to its uniform structure, good biocompatibility, and the ability to initiate the cellular response to form apatite, the major inorganic component of bone. These mainstream views always lack sufficient conviction to clarify the causes and mechanism of bone formation. Mechanical cues sensed by the cells are propagated and transduced into signaling cascades, which lead to transient or sustained cellular responses. Besides, matrix stiffness is recognized as a potent regulator to alter the epigenetic status of cells during bone regeneration.^[^ ^10^ ^]^ A recent study uncovered the role of translocation 2 (Tet2) in response to matrix stiffness, and Tet2‐induced DNA demethylation can be promoted with mechanical cues from stiff matrix.^[^ ^11^ ^]^ Understanding the cellular response to matrix stiffness would help us to realize hydrogels with optimal combinations of structure manipulability, bioactivity, and clinical efficacy. Thus, developing and mechanistically investigating a gelling system with structural controllability and excellent mechanical properties is particularly appealing.

**Figure 1:**
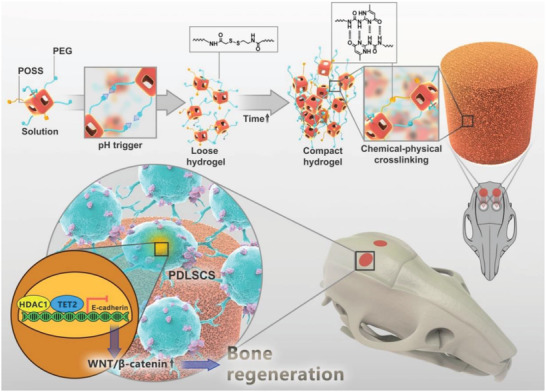
Schematic of the dual‐crosslinked gelling system and its tunable architecture and mechanical properties, which promote PDLSC‐mediated bone regeneration by activating WNT/*β*‐catenin through TET2 epigenetic regulation.

Herein, we developed a class of dual crosslinked gelling systems with tunable architecture and mechanical properties to promote stem cells’ osteogenic capacity (**Figure** **1**). The hydrogel precursor, POSS‐P_6_‐U_2_, features a disulfide‐linked core/shell structure in which POSS acts as a rigid core surrounded by six disulfide‐linked flexible hydrophilic polyethylene glycol (PEG) chains that form the shell and two UPy groups. The thiol‐disulfide exchange reaction was employed to fabricate a “living” in situ chemical network because of its pH‐responsive “on/off” functionality, while the self‐complementary UPy motif reinforces the local microstructure to enhance mechanical properties. More importantly, this multifunctional POSS unit anchoring into the network possessed favorable cytocompatibility and osteogenic bioactivity that could support cell attachment, spreading, and proliferation. In this work, we demonstrated that the osteogenic differentiation potential of seeded periodontal ligament stem cells (PDLSCs) increased with suitable matrix stiffness. PDLSCs are one type of dental‐derived mesenchymal stem cells, attractive for craniofacial regeneration applications as they might be more committed to differentiating into craniofacial tissues compared with none dental‐derived stem cells under certain extracellular stimuli.^[^ ^12^ ^]^ Mechanistically, we have shown that the key epigenetic regulator TET2, which is associated with HDAC1, is upregulated in PDLSCs seeded on a stiff substrate, leading to suppression of E‐cadherin transcription and WNT/*β*‐catenin activation, thus promoting osteogenesis in the PDLSCs. Our findings demonstrate the potential clinical application of the POSS‐UPy hybrid hydrogel and have identified matrix stiffness as a key epigenetic regulator of PDLSCs‐mediated bone regeneration.

## 2 Results and Discussion

**Figure 2:**
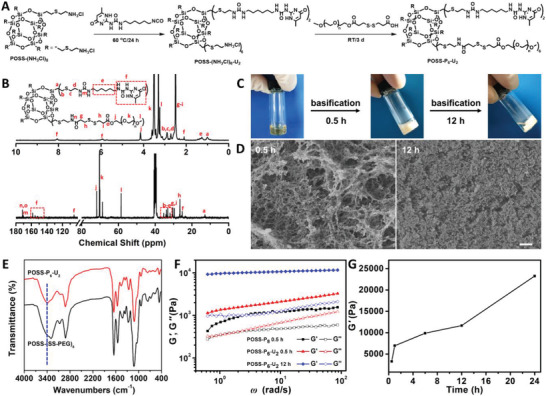
Synthesis and characterization of the POSS‐P_6_‐U_2_ hydrogel system. A) Synthetic route and B) ^1^H NMR and ^13^C NMR spectra of POSS‐P_6_‐U_2_ polymer. C) Schematic representation and D) SEM images of POSS‐P_6_‐U_2_ solution and transformation methodology after basification for 0.5 and 12 h. Scale bars: 1 µm. E) FTIR spectra of POSS‐(SS‐PEG)_8_ and POSS‐P_6_‐U_2_ hydrogels after basification for 12 h. F) Storage modulus (G΄) and loss modulus (G″) of POSS‐(SS‐PEG)_8_ and POSS‐P_6_‐U_2_ solutions after basification for 12 h as a function of strain (*γ*). G) Storage modulus variation of POSS‐P_6_‐U_2_ solution after basification as a function of time (n = 3 images per group).

In view of the high hydrophobicity of POSS unit and UPy moiety, we employed the POSS‐P_6_‐U_2_ polymer as the precursor molecule for the construction of tailored hybrid hydrogel through facile two‐step reactions (**Figure** **2**A). POSS was designated as the model core because of its rigid cage‐like structure, excellent thermal, mechanical stabilities, and favorable osteogenic effects. UPy units and PEG as various shell moieties were doped into well‐defined POSS by reaction of amino with isocyanate and amidation (Figure S1A,S1B, Supporting Information). ^1^H NMR analysis (Figure 2B) showed the typical proton peaks of PEG chains (3.3–4.2 ppm) and UPy components (1.0–1.5 ppm) with the approximate integral area ratio (*I*_a_/*I*_b_/*I*_c_ = 1:6:1), and ^13^C NMR spectrum also provided the evidence to verify the successful preparation of targeted POSS‐P_6_‐U_2_ polymer. It is mentioned that since the gel precursor of POSS‐P_6_‐U_2_ may not be a precise macromolecule and has three isomer structures, but actually, their differential components and various stereoisomers have little influence on the controlled network once the gel formation is initiated, because a variety of interchangeable PEG chain conformations can curl and entangle with each other to facilitate the hydrophobic areas, forming a series of random heterogeneous aggregates in solutions, let alone the more complex sol‐gel transition and full‐scale crosslinking evolution process.^[^ ^13^ ^]^

This amphiphilic polymer has high chemical stability during storage and good solubility in water. When a 10 wt.% polymer solution was basified to pH 12, a few thiols were first converted to thiolates that subsequently triggered the thiol‐disulfide exchange reaction, which led to cross‐linking of the POSS cores and release of the reassembled shells. The PEG shell has good solubility in water and can be regarded as a good leaving group, and the strongly associating UPy motif can slowly form a quadruple hydrogen bonding to construct the physical interaction, so the driving force was due to the shifting of the equilibrium toward the stable cross‐linked products through the release of the PEG shells and linkage of cross‐linked junction. With a greater extent of exchange and dynamic evolution, more PEG segments were released and a higher degree of cross‐linking was achieved as well as the construction of strong hydrogen bond interactions. Therefore, a white loose hydrogel (POSS‐P_6_‐U_2_‐0.5 h) was formed around 0.5 h later and further shrank into a compact hydrogel (POSS‐P_6_‐U_2_‐12 h) after 0.5 day of aging (Figure 2C). It is mentioned that the transition from the loose to compact hydrogels can be interrupted at any point in time by neutralization and restarted by re‐basification with an “on/off” function. Notably, on account of the important contribution of self‐complementary hydrogen bonding between UPy motifs, the physical network was gradually built and dynamically evolved to incessantly optimize the network microstructure and improve the hydrogel mechanics. In this case, a dual cross‐linked (DC) gelling system is synergistically regulated that can simultaneously solve the issues of structure tunability and mechanics optimization. Scanning electron microscope (SEM) (Figure 2D) depicted the hydrogels with significantly different architectures and showed that a longer aging time facilitated the formation of a more compact hydrogel with a smaller pore size and denser structure. While the vibration at 3426 cm^−1^ presented in the IR spectrum of the as‐prepared POSS‐P_6_‐U_2_ ascribed to strongly hydrogen‐bonded urea carbonyls and hydrogen‐bonded between the pyrimidinone carboxylate and urea (Figure 2E and Figure S2, Supporting Information).

For a clear demonstration of the mechanical property, rheological measurements were performed to obtain the storage modulus (G΄) and loss modulus (G″). The introduction of POSS units provided high strength for the hydrogels and the higher G΄ than G″ implied the typical formation of stable hydrogels (Figure 2F). Compared to the single chemical hydrogel from POSS‐(SS‐PEG)_8_ 0.5 h (POSS‐P_8_‐0.5 h), DC hydrogel from POSS‐P_6_‐U_2_‐0.5 h exhibited a higher mechanical strength originating from the robustly physical networks, which enabled hydrogels to dissociate to dissipate energy upon the applied strain. With the increase in crosslinking time, the microstructure of hybrid hydrogels became more compact and the swelling rate was lower due to the reduction of hydrophilicity by dissociation of hydrophilic PEG shells and optimization of the hydrogel network by self‐complementary UPy components. During this gelation process, a multistage abrupt increase of the storage modulus G΄ implied the formation of the stable hydrogel with high strength (Figure 2G), however, the longer crosslinking time could lead to a decrease in gel toughness due to the high density of inorganic POSS aggregates. In addition, these as‐obtained hybrid hydrogels were biodegradable via the cleavage of disulfide linkages.^[^ ^6^ ^]^ Therefore, stable hybrid hydrogels with customized structures and properties can be easily produced through control of the system pH within a predetermined time.

**Figure 3:**
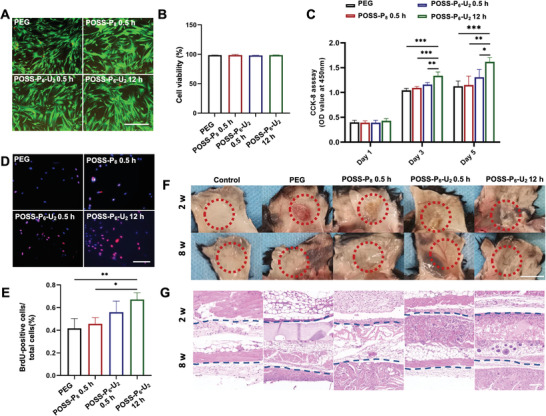
Biocompatibility and degradation rates of the POSS‐P_6_‐U_2_ hydrogels. A) Live/dead staining and B) semi‐quantitative analysis showing the viability of the PDLSCs on different hydrogel substrates. Scale bars: 200 µm. C) CCK‐8 analysis indicating cell viabilities on the POSS‐P_6_‐U_2_‐0.5 h and POSS‐P_6_‐U_2_‐12 h hydrogels are significantly higher than those of the other groups. D) BrdU and E) semi‐quantitative analysis revealing the proliferation rate of PDLSCs is significantly higher on the POSS‐P_6_‐U_2_‐12 h hydrogel. Scale bars: 100 µm. F) The gross view and G) histological analysis of the hydrogel implant areas in C57BL/6J mice 2 and 8 weeks after implantation. The dotted line represents the boundary between the tissue and the implants. Scale bars: 100 µm (F). Scale bars: 1 cm (G). (n = 3 per group; *p < 0.05, **p < 0.01, ***p < 0.001)

To investigate the biocompatibility of the hybrid hydrogels for further biomedical applications, PDLSCs were co‐cultured with hydrogels and analyzed with the live/dead staining and Cell Counting Kit‐8 (CCK‐8) assay. Live/Dead staining confirmed that most of the PDLSCs cultivated in the hydrogels were alive (close to 100%) (**Figure** **3**A), and no significant difference between groups was detected (Figure 3B). Then, CCK‐8 assays proved the low cytotoxicity of the hydrogels (Figure 3C). Tetra‐PEG, POSS‐P_8_‐0.5 h, POSS‐P_6_‐U_2_‐0.5 h, and POSS‐P_6_‐U_2_‐12 h hydrogels showed similar cell viability on day 1. As time passed, the number of viable cells cultured on different hybrid hydrogels was elevated. The level of cell viability on the POSS‐P_6_‐U_2_‐0.5 h and 12 h hydrogels were significantly higher than the tetra‐PEG and POSS‐P_8_‐0.5 h groups on different time points, and the POSS‐P_6_‐U_2_‐12 h hydrogels demonstrated the highest cell viability among all the groups. In addition, Bromodeoxyuridine (5‐bromo‐2′‐deoxyuridine, BrdU) analyses were also carried out to investigate the cell proliferation rate on hydrogels (Figure 3D). After incubation for 48 h, the percentage of BrdU positive PDLSCs was significantly increased in POSS‐P_6_‐U_2_‐12 h group (*p* < 0.01, Figure 3E), which was consistent with the result of CCK‐8 analysis above. Furthermore, we implanted the hydrogels subcutaneously in mice to validate their in vivo degradation behavior. The size of all the hybrid hydrogels decreased significantly from 2 to 8 weeks post‐implantations, as indicated by the gross observation (Figure 3F). The hematoxylin and eosin (H&E) staining showed the scaffold structures of hybrid hydrogels were collapsed and a few residuary hydrogels were detected at the implanted sites after 8 weeks of implantation in POSS‐P_8_‐0.5 h, and POSS‐P_6_‐U_2_‐12 h groups, exhibiting the slower degradation profiles of hybrid hydrogels compared with tetra‐PEG hydrogel in vivo (Figure 3G and Figure S3A, Supporting Information). Interestingly, we found that the POSS‐P_6_‐U_2_‐0.5 h hydrogel displayed an equal degradation rate as the tetra‐PEG group. On account of the hydrophobic nature of POSS units and dense networks of POSS‐incorporated hybrid hydrogels, we speculated that initial degradation may be related to the high swelling behavior of hybrid hydrogels and the dissociation of quadruple hydrogen bonding, but once the degradation proceeded, the covalent bonding of hydrophobic POSS units would increase the crosslinking density to prevent the enzymes from diffusion into the interior of hydrogels, offering a hydrophobic barrier to prolong its period of hydrolysis and enzymolysis. In addition, the non‐alkaline environment in the implanted region also stabilized the disulfide bonds to slow down degradation time. In this case, the remaining modulus of the hybrid hydrogel after 8 weeks can provide long‐term support for bone regeneration. To evaluate the host response to the implanted hydrogels, we used CD11b, a macrophage‐specific surface marker, to detect the hydrogel implanted sites. Figure S3B,S3C, Supporting Information, shows an increased level of CD11b positive cells in the hydrogel implantation sites when compared with non‐implantation control group. No significant difference was detected between tetra‐PEG, POSS‐P_8_‐0.5 h, POSS‐P_6_‐U_2_‐0.5 h, and POSS‐P_6_‐U_2_‐12 h groups at the same time point. As time passed, the ratio of CD11b positive cells decreased, indicating a low level of macrophage‐mediated degradation at 8 weeks post‐implantations. The inflammation level around the hydrogel implanted sites among all hydrogel groups was milder with fewer detection of interferon‐*γ* (IFN‐*γ*) or tumor necrosis factor‐*α* (TNF‐*α*) positive inflammatory cells at 8 weeks compared with 2 weeks (Figure S3D–S3G, Supporting Information). These results indicated this hybrid hydrogel system possessed a great biocompatible potential, with the capacity of degradation in vivo, supporting cell growth and low cytotoxicity.

**Figure 4:**
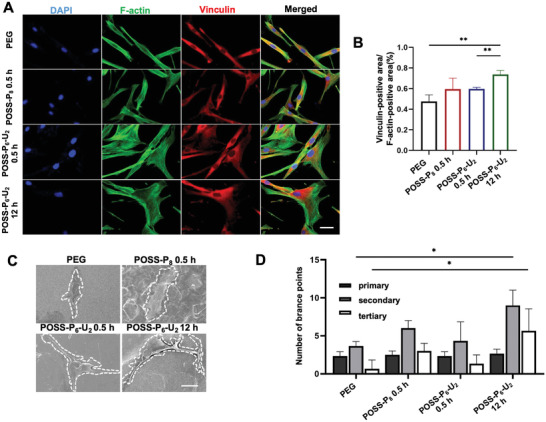
Effect of POSS‐P_6_‐U_2_ hydrogel on cell attachment and morphology. A) Immunofluorescence staining showing the cytoskeletons, stress fibers, and FAPs located at the ends of the stress fibers inside the PDLSCs for all the hydrogels. Scale bars: 50 µm. B) Semi‐quantitative analysis revealing that POSS‐P_6_‐U_2_‐12 h hydrogel group exhibits the highest FAP level. C) SEM images of PDLSCs morphology after cultivation on different hydrogels for 48 h. Scale bar: 50 µm. PDLSCs morphology was analyzed to determine D) the number of primary, secondary, and tertiary branch points. (n = 3 per group; *p < 0.05, **p < 0.01)

Cell behaviors can be influenced by the substrates with different surface stiffness and might further determine cells fate, POSS‐P_6_‐U_2_‐12 h with high mechanical strength exhibited a better support for cell attachment, validated by vinculin for focal adhesion points (FAPs) staining and F‐actin for cytoskeleton labeling (**Figure** **4**A). The PDLSCs on all hydrogels formed clear cytoskeleton and stress fibers. Meanwhile, the highest level of FAPs was detected in the PDLSCs cultured on POSS‐P_6_‐U_2_‐12 h substrate, mainly located at the ends of the stress fibers (Figure 4B). As McBeath et al. suggested that adherent, flatter, and well‐spread PDLSCs tended to osteogenesis, while less adherent, rounded cells undergo adipogenesis.^[^ ^14^ ^]^ SEM morphological observation was used to detect the influence of POSS‐P_6_‐U_2_ hydrogels on PDLSCs morphology (Figure 4C). We found that the adherent PDLSCs on the PEG substrate were elongated fibroblast‐like and spindle‐shaped in appearance, whereas the PDLSCs seeded on POSS‐P_6_‐U_2_‐12 h substrate became the typical highly branched osteoblast‐like morphology. The analysis of cell branching numbers demonstrated that the PDLSCs on POSS‐P_6_‐U_2_‐12 h hydrogels significantly displayed most branching than the control group (Figure 4D).

**Figure 5:**
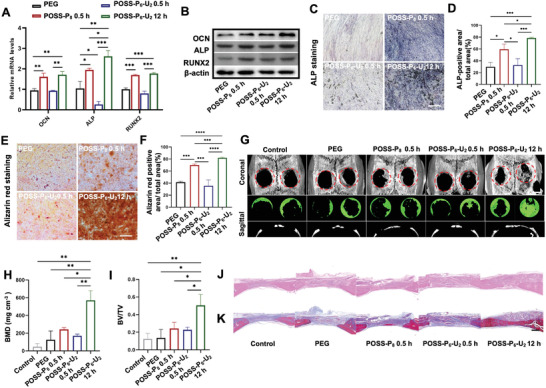
POSS‐P_6_‐U_2_ hydrogel regulates the osteogenic differentiation of PDLSCs in vitro and in vivo. A) qPCR quantification and B) Western blotting analysis showing the highest expression of osteogenic markers (RUNX2, OCN, and ALP) in the POSS‐P_6_‐U_2_‐12 h hydrogels. C) ALP staining and the D) positive area percentages, E) ARS staining, and the F) positive area percentages revealing POSS‐P_6_‐U_2_‐12 h hydrogels significantly enhanced osteogenic differentiation of PDLSCs. G) 3D reconstruction of µCT images of new bone formation in rat cranium at 8 weeks after PDLSCs/hydrogels implantation, revealing the greatest abundant bone regeneration in the POSS‐P_6_‐U_2_‐12 h group. Scale bars: 5 mm. Quantitative analysis of H) BMD and I) bone volume/tissue volume (BV/TV) of regenerated bone tissue. J) H&E staining and K) Masson's trichrome staining at 8 weeks after implantation showing the same trends in bone regeneration as those depicted by the µCT analysis. Scale bars, 500 µm. (n = 3 per group; *p < 0.05, **p < 0.01, ***p < 0.001)

It has been reported that morphology is tightly coupled with cells’ growth and function.^[^ ^15^ ^]^ Highly branched cell morphologies with extended pseudopodia are capable of inducing osteogenesis.^[^ ^16^ ^]^ Thus, to confirm the osteo‐induction potential of POSS‐P_6_‐U_2_‐12 h substrates on PDLSCs, runt‐related transcription factor 2 (RUNX2), alkaline phosphatase (ALP), and osteocalcin (OCN) were used as osteogenesis markers and analyzed with qPCR assay (**Figure** **5**A) and Western blots (Figure 5B). Interestingly, we found that after incubation for 7 days, the PDLSCs cultured in POSS‐P_8_‐0.5 h and POSS‐P_6_‐U_2_‐12 h hydrogels expressed higher levels of osteogenesis markers than that of tetra‐PEG and POSS‐P_6_‐U_2_‐0.5 h groups, meanwhile POSS‐P_6_‐U_2_‐12 h hydrogels displayed the highest osteo‐inductive capacity among all the experiment groups both on RNA and protein levels. The PDLSCs cultured in POSS‐P_6_‐U_2_‐0.5 h and tetra‐PEG hydrogels displayed relative comparative osteogenesis potential. Next, ALP (Figure 5C,D) and alizarin red staining (ARS) (Figure 5E,F) were also used to verify the osteo‐induction capacity of hydrogels on days 7 and 14 respectively, the results were consistent with the qPCR and Western blot analysis. These results indicated that POSS‐P_6_‐U_2_‐12 h hydrogels, with higher mechanical strength and stiffness from the robustly physical‐chemical networks, obtained superb osteogenic induction potential, which might be a promising scaffold for bone tissue engineering.

In addition to the verify the effect of POSS‐P_6_‐U_2_ hydrogels on the PDLSCs osteogenesis in vitro, we next investigated whether these hybrid hydrogels combined with PDLSCs could promote bone regeneration in vivo. PDLSCs in different hybrid hydrogels were implanted into freshly established rat critical sized cranial bone defects. After post‐transplantation for 8 weeks, microcomputed tomography (µCT) images (Figure 5G) illustrated that the PDLSCs/POSS‐P_6_‐U_2_‐12 h hydrogel group obtained the largest amount of new bone formation. Fewer bone formation occurred after the delivery of PDLSCs in tetra‐PEG, POSS‐P_8_‐0.5 h, and POSS‐P_6_‐U_2_‐0.5 h hydrogels. Quantitative analysis revealed the highest bone mineral density (BMD) (Figure 5H) and bone volume (BV/TV) (Figure 5I) in the PDLSCs/POSS‐P_6_‐U_2_‐12 h hydrogel group (**p < 0.01). Moderately increased new bone area, BV/TV, and BMD were revealed in POSS‐P_8_‐0.5 h hydrogel group. While the least bone regeneration was shown in the tetra‐PEG group, POSS‐P_6_‐U_2_‐0.5 h, and control group. In accordance with µCT analysis, histological analysis with H&E (Figure 5J and Figure S4A,S4B, Supporting Information) and Masson's trichrome staining (Figure 5K and Figure S4C, Supporting Information) showed an increased bone formation in PDLSCs/POSS‐P_6_‐U_2_‐12 h hydrogel and PDLSCs/POSS‐P_8_‐0.5 h hydrogel groups compared to PDLSCs/PEG hydrogel and PDLSCs/POSS‐P_6_‐U_2_‐0.5 h hydrogel groups, wherein PDLSCs/POSS‐P_6_‐U_2_‐12 h hydrogel group revealed the most efficient promotion on new bone formation. The percentage of OCN‐positive cells detected by immunofluorescence staining around the implantation area showed the same trend (Figure S4D,S4E, Supporting Information). To further evaluate the inflammation level of the implantation sites, we used immunofluorescence staining to detect the levels of IFN‐*γ* and TNF‐*α*, no obvious inflammation was found in PDLSCs/hydrogel implantation groups compared with control group (Figure S4F–S4H, Supporting Information). To further investigate if the osteo‐induction effect is determined by the high strength of POSS‐P_6_‐U_2_ hydrogels system, instead of the pure UPy moiety, we cultivated the PDLSCs on tetra‐PEG‐UPy and used tetra‐PEG as control. The ARS staining indicated that the osteo‐inductive effect of both of the groups showed no significant difference (Figure S5A, Supporting Information). Next, we also compared the in vivo bone regeneration capacity of PEG‐UPy with PEG hydrogel, and found no significant difference between two groups by using µCT (Figure S5B–S5D, Supporting Information) and histological analysis (Figure S5E,S5F, Supporting Information), which further confirmed the osteo‐induction effect is significantly determined by the controlled cross‐linked hydrogen‐bonded POSS‐P_6_‐U_2_ system with synergistic regulation of pH‐responsive chemical networks and robust physical networks with the dynamic self‐optimized arrangement, instead of the pure UPy moiety.

It was mentioned that although POSS‐P_6_‐U_2_‐12 h exhibited superior osteogenesis, POSS‐P_8_‐0.5 h showed a more pronounced osteogenesis activity than that of POSS‐P_6_‐U_2_‐0.5 h. Based on Figure S5, Supporting Information, we demonstrated the UPy motif itself has no effect on the osteogenesis, which is employed to optimize the microstructure and enhance mechanical properties via the hierarchical hydrogen‐bonding interactions. For the POSS‐P_8_‐0.5 h, we previously found a dynamic and tunable manipulation of sequential morphology and size transformation in situ via a hierarchical assembly strategy based on a living thiol‐disulfide exchange reaction.^[^ ^4^ ^]^ By tailoring the external stimuli in an alkaline environment, the reactive points can be generated at the ends of initially unimolecular micelles, which subsequently drive the pre‐assemblies to periodically proceed into the hierarchically micellar connection, axial growth, bending, and cyclization processes from nanoscopic solution aggregate to macroscopic particles. This axially oriented structure facilitates cell differentiation and growth,^[^ ^17^ ^]^ but does not have enough flexible chains for energy dissipation, exhibiting brittle and fragile behaviors. In contrast, on the account of the weak self‐complementary effect of the UPy motif within a short time, POSS‐P_6_‐U_2_‐0.5 h exhibits a little higher modulus but spoils the ordered arrangement of structures at the same time, which may lead to the inferior osteogenesis activity than that of POSS‐P_8_‐0.5 h. However, extending the crosslinking time to 12 h, sufficient self‐adaptive behaviors endow the hydrogel with a newly well‐ordered network and excellent mechanics and bioactivity for osteogenesis.

**Figure 6:**
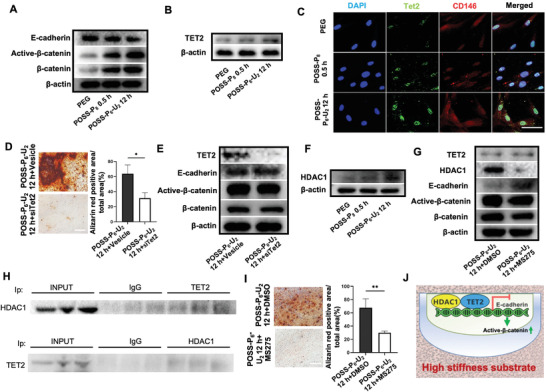
Epigenetic regulatory mechanism of high strength hydrogels in promoting PDLSCs osteogenesis through Tet2/HDAC1/E‐cadherin/*β*‐catenin signaling cascade. A) Western blotting analysis indicating downregulated levels of E‐cadherin proteins and upregulated levels of Active‐*β*‐catenin and *β*‐catenin in PDLSCs/POSS‐P_6_‐U_2_‐12 h hydrogel groups. B) Western blotting analysis showing upregulated levels of Tet2 in PDLSCs on the POSS‐P_6_‐U_2_‐12 h hydrogels compared with tetra‐PEG and POSS‐P_8_‐0.5 h hydrogels. C) Immunofluorescence analysis showing the MSCs marker CD146 was co‐expressed with Tet2 in PDLSCs. Scale bars: 50 µm. D) ARS staining and the positive area percentages revealing decreased osteogenic differentiation of PDLSCs after knockdown of Tet2. Scale bars: 500 µm. E) Western blotting analysis verifying upregulated levels of E‐cadherin and downregulated levels of Active‐*β*‐catenin and *β*‐catenin proteins after effective knockdown of Tet2. F) Western blotting analysis showing upregulated levels of HDAC1 proteins in PDLSCs on the POSS‐P_6_‐U_2_‐12 h hydrogels compared to tetra‐PEG and POSS‐P_8_‐0.5 h hydrogels. G) Western blotting analysis revealing an upregulation of E‐cadherin expression, a downregulation of Active‐*β*‐catenin expression, and no specific effect on Tet2 expression after effective knockdown of HDAC1. H) CoIP results demonstrating that HDAC1 and Tet2 could associate reciprocally. I) ARS staining and the positive area percentages showing declined osteogenic differentiation of PDLSCs after the knockdown of HDAC1. Scale bars: 500 µm. J) A schematic representation of molecular signaling that mediates stiff substrate‐induced PDLSCs osteogenesis. (n = 3 per group; *p < 0.05, **p < 0.01)

Mechanical strength affects the MSCs potency through several cell/matrix and cell/cell interactions, including stimulation of integrin and cadherin receptors.^[^ ^18^ ^]^ It has been reported that mechanical stress can inhibit cadherin, allowing the *β*‐catenin translocated into the nucleus and initiating osteogenesis‐related gene transcription.^[^ ^19^ ^]^ Activation of WNT/*β*‐catenin signaling pathway is essential for bone tissue regeneration and cell adhesion.^[^ ^20^ ^]^ By using western blot analysis, we found that PDLSCs cultured with high strength POSS‐P_6_‐U_2_‐12 h hydrogel expressed a lower level of E‐cadherin, accompanied by promoted expression levels of active‐*β*‐catenin and *β*‐catenin (**Figure** **6**A). Meanwhile, as the co‐cultivate time passed, the expression levels of active‐*β*‐catenin and *β*‐catenin increased continually, followed by the decreased level of E‐cadherin (Figure S6A,S6B, Supporting Information), thus resulting in the activation of PDLSCs‐mediated bone regeneration.

Epigenetics is defined as heritable changes without variation in DNA sequence and plays a vital role in regulating the fate of MSCs.^[^ ^21^ ^]^ It has been well appreciated that highly functionalized biomaterials could change the epigenetic status of MSCs and ultimately promote their osteogenic differentiation.^[^ ^22^ ^]^ Tet family participated in maintaining MSCs functions in the reported literatures,^[^ ^23^ ^]^ while Tet2 overexpression could promote osteogenesis of MSCs.^[^ ^24^ ^]^ Moreover, a recent study uncovered the role of Tet2 in response to matrix stiffness, and Tet2‐induced DNA demethylation can be promoted with mechanical cues from stiff matrix.^[^ ^11^ ^]^ To investigate how POSS‐P_6_‐U_2_‐12 h hydrogel inhibited E‐cadherin, we verified the Tet2 expression levels in the PDLSCs co‐cultured with hydrogels, and found that Tet2 was significantly elevated in the PDLSCs cultured in high strength POSS‐P_6_‐U_2_‐12 h hydrogel compared with tetra‐PEG and POSS‐P_8_‐0.5 h groups (Figure 6B). Immunostaining confirmed that MSCs marker CD146 was co‐expressed with Tet2 in PDLSCs (Figure 6C). Next, to evaluate if Tet2 regulates POSS‐P_6_‐U_2_‐12 h hydrogel‐induced osteogenesis, we used siRNA to downregulate Tet2 expression level of hydrogel‐treated PDLSCs, and found a significant decrease of osteo‐inductive capacity on POSS‐P_6_‐U_2_‐12 h hydrogel using ARS (Figure 6D) and ALP staining (Figure S6C, Supporting Information). Furthermore, Western blot was used to confirm if E‐cadherin and *β*‐catenin were the downstream targets of Tet2, which revealed that E‐cadherin was significantly elevated and active‐*β*‐catenin/*β*‐catenin were decreased in Tet2 siRNA treated PDLSCs (Figure 6E). Collectively, these results indicated the high strength of POSS‐P_6_‐U_2_‐12 h hydrogel had capacities to promote PDLSCs osteogenesis by increasing Tet2 expression level, thereby inhibiting the E‐cadherin and continuously promoting the WNT/*β*‐catenin signaling activation.

Tet plays a dual role as an epigenetic modulator to control gene transcription, which can recruit repressor complexes, such as histone deacetylases (HDACs), enabling them to regulate gene expression independent of DNA demethylation process.^[^ ^25^ ^]^ To further evaluate whether E‐cadherin is a direct target of Tet2, we analyzed the expression level of HDAC1 by using western blot (Figure 6F) and immunostaining (Figure S6D, Supporting Information), and the up‐regulated expression level of HDAC1 was detected in the PDLSCs cultured with POSS‐P_6_‐U_2_‐12 h hydrogel, which was consistent with Tet2. To investigate whether HDAC1 is recruited by Tet2 and verify its role in the process of POSS‐P_6_‐U_2_‐12 h hydrogels promoting osteogenic differentiation, we used MS275 to inhibit HDAC1 expression in PDLSCs treated with POSS‐P_6_‐U_2_‐12 h hydrogels. Western blot analysis indicated that after inhibition of HDAC1, the expression levels of E‐cadherin increased significantly, and active‐*β*‐catenin/*β*‐catenin decreased accordingly (Figure 6G). The semi‐quantitative analysis of the western blots in Figure 6 is provided in Figure S7, Supporting Information. In addition, a coimmunoprecipitation (CoIP) assay was employed to confirm the recruitment of HDAC1 by Tet2. Figure 6H shows that HDAC1 and Tet2 could associate reciprocally. Through associating with HDAC1, Tet2 epigenetically inhibited the transcriptional expression of E‐cadherin, meanwhile, continuously activating the WNT/*β*‐catenin signaling pathway. Next, inhibition of HDAC1 in PDLSCs resulted in decreased osteogenesis induced by POSS‐P_6_‐U_2_‐12 h hydrogels. The ARS (Figure 6I) and ALP staining (Figure S6E, Supporting Information) results confirmed the osteo‐induction process of POSS‐P_6_‐U_2_‐12 h hydrogels was significantly blocked. Further test on the tissue level, we used immunofluorescence staining to detect the levels of Tet2 in the bone regeneration site of cranial bone defects model, and revealed the percentage of Tet2 positive cells is relatively high in PDLSCs/POSS‐P_6_‐U_2_‐12 h hydrogel implantation groups compared with others (Figure S8, Supporting Information). These results revealed a potential epigenetic regulatory mechanism of high‐strength hydrogels in promoting PDLSCs osteogenesis differentiation through Tet2/HDAC1/E‐cadherin/*β*‐catenin signaling cascade (Figure 6J). More examinations could be further examined in vivo to confirm the high stiffness cues in regulating Tet2‐mediated bone regeneration in the future.

## 3 Conclusion

In conclusion, we have demonstrated a dual crosslinked gelling system from a precursor of POSS‐P_6_‐U_2_ polymer with customized microstructures and optimal mechanical properties to control PDLSCs osteogenic capacity in vitro and in vivo. Depending on synergistic regulation of pH‐responsive chemical networks with an “on/off” function and physical networks with the dynamic self‐optimized arrangement, thiol‐disulfide exchange reaction endowed hydrogels with controlled architectures while UPy moieties contributed to a prominent increase in mechanical strengths. More intriguingly, the controllable POSS‐P_6_‐U_2_‐12 h hydrogel with a higher matrix stiffness displayed a significant increment of osteo‐induction ability toward PDLSCs. Mechanistically, we first found that the key epigenetic regulator TET2 was elevated after PDLSCs seeding on the stiffer substrates, associated with HDAC1, leading to the suppression of E‐cadherin transcription and promoting Wnt/*β*‐catenin sustained activation, which resulted in the elevated osteogenesis capacity of PDLSCs. Therefore, our findings demonstrated the potential clinical application of the PEG‐POSS‐UPy hybrid hydrogel, and uncovered the matrix stiffness as a key epigenetic regulator of PDLSCs mediated bone regeneration, which will hold great potential in the rational design of controlled gelling systems and motivate scientists to fabricate more advanced materials for various high‐tech applications.

## 4 Experimental Section

Detailed experimental section can be found in the Supporting Information

## Conflict of Interest

The authors declare no conflict of interest.

## Supporting information

Supporting Information

## Data Availability

The data that support the findings of this study are available on request from the corresponding author. The data are not publicly available due to privacy or ethical restrictions.
