# Supplementary material for: Mechanically Robust Hydrogels Facilitating Bone Regeneration through Epigenetic Modulation
Source: Adv Sci (Weinh). 2022 Sep 25;9(32):2203734. doi: 10.1002/advs.202203734 (PMC9661832; doi:10.1002/advs.202203734)
Supplement: Supplementary file 1 — Supporting Information [file ADVS-9-2203734-s001.pdf]

Supporting Information

Mechanically Robust Hydrogels Facilitating Bone Regeneration through Epigenetic Modulation

Tingting Yu†, Lingyun Zhang†, Xueyu Dou†, Rushui Bai, Hufei Wang, Jie Deng, Yunfan Zhang, Qiannan Sun, Qian Li, Xing Wang, Bing Han**

Materials

Octavinyl POSS (98%, Hybrid Plastics), cysteamine hydrochloride (98%, Energy Chemical), mercapto-poly(ethylene glycol) (PEG-SH, M_n = 750 g mol^{-1}) and octa (3-mercaptopropylsilsesquioxane) (POSS-(SH)$_8$) were synthesized according to our previous work [1]. 2, 2'-dithiodipyridine (99%, J&K), 2,2′-dimethoxy-2-phenylacetophenone (99%, J&K), 2-amino-4-hydroxy-6-methylpyrimidine (98%, TCI), 1,6-diisocyanatohexane (98%, TCI), *N*,*N*′-dicyclohexylcarbodiimide (98%, Energy Chemical), *N*-hydroxysuccinimide (98%, Energy Chemical), 1-ethyl-3-(3-dimethylaminopropyl) carbodiimide hydrochloride (99%, Energy Chemical), 4-dimethylaminopyridine (99%, J&K), 3,3′-dithiodipropionic acid (99%, TCI), sodium hydroxide (96%, Beijing Chemical Works), dimethyl sulfoxide (SuperDry, 99.7%, J&K), triethylamine (Extra Dry, 99.5%, Energy Chemical). Tetrahydrofuran (THF) and dichloromethane (DCM) were dried according to the typical procedures. Deionized water, methanol (MeOH), ether and *n*-hexane were used directly.

For immunofluorescence staining, polyclonal rabbit anti-Tet2 antibody (ab245287, 1:200 diluted) and monoclonal rabbit anti-CD146 antibody (ab75769, 1:200 diluted) were purchased from Abcam, USA; monoclonal mouse anti-HDAC1 antibody (sc-81598, 1:50 diluted), polyclonal mouse anti-CD90 antibody (sc-53116, 1:50 diluted), and polyclonal goat anti-IFN-γ (sc-1377, 1:50 diluted) were purchased from Santa Cruz Biotechnology, USA; polyclonal rabbit anti-OCN antibody (DF12303, 1:200 diluted) was purchased from

affbiotech, China; FITC-Phalloidin (CA1620, 1:500 diluted), goat anti-rabbit IgG/RBITC (SR134, 1:200 diluted), goat anti-mouse IgG/RBITC (SF131, 1:200 diluted), and goat anti-mouse IgG/FITC (SF131, 1:200 diluted) were purchased from Solarbio, China); rabbit anti-goat IgG/FITC (ZF-0314, 1:200 diluted) was purchased from ZSGB-BIO, China); monoclonal mouse anti-vinculin antibody (V9264, 1:200 diluted) were purchased from MilliporeSigma, USA.

For Western blotting, monoclonal mouse anti-RUNX2 antibody (sc-101145, 1:200 diluted), monoclonal mouse anti-OCN antibody (sc-376726, 1:200 diluted), monoclonal mouse anti-ALP antibody (sc-365765, 1:200 diluted), monoclonal mouse anti-E-cadherin antibody (sc-8428, 1:200 diluted), monoclonal mouse anti-HDAC1 antibody (sc-81598, 1:200 diluted), and monoclonal mouse anti-β-catenin antibody (sc-7963, 1:200 diluted) were purchased from Santa Cruz, USA; polyclonal rabbit anti-Tet2 antibody (ab245287, 1:1000 diluted) and polyclonal rabbit anti-active-β-catenin antibody (ab246504, 1:1000 diluted) were purchased from Abcam, USA; goat anti-mouse IgG/HRP (SE131, 1:10000 diluted) and goat anti-rabbit IgG /HRP (SE134, 1:10000 diluted) were purchased from Solarbio, China； and monoclonal mouse anti-β-actin antibody (A1978, 1:200 diluted) was purchased from Sigma, USA.

For the COIP studies, normal rabbit IgG (2729S) and Tet2 antibody (45010s) were purchased from Cell Signalling, USA; ProteinIso® Protein A/G Resin (DP501) was purchased from TransGen Biotech, China; and monoclonal mouse anti-HDAC1 antibody (sc-81598) was purchased from Santa Cruz, USA.

For the inhibition studies, Entinostat (MS275) was purchased from MedChem Express, USA, Tet2 siRNA (sc-88934) and Control siRNA (sc-37007) was purchased from Santa Cruz, USA.

ALP staining was performed using BCIP/NBT Alkaline Phosphatase Color Development Kit (Beyotime, China). ASR staining was performed using Alizarin red S (Sigma-Aldrich, USA).

Synthesis of POSS-(NH_3Cl)$_8$

Octavinyl POSS (4.00 mmol, 2.53 g), cysteamine hydrochloride (38.5 mmol, 4.37 g) and 2,2′-dimethoxy-2-phenylacetophenone (1.32 mmol, 0.34 g) were dissolved in 50 mL of THF/MeOH (v/v = 3/1). The mixture was irradiated under 365 nm UV lamp at room temperature for 6 h under nitrogen protection. A mount of white precipitate was collected by filtration and extensively washed with THF/MeOH (v/v = 5/1). The product was dried under vacuum overnight to obtain white powder with the yield of 92.3%.

Synthesis of UPy-NCO

2-amino-4-hydroxy-6-methylpyrimidine (10.00 mmol, 1.25 g) and 1,6-diisocyanatohexane (60.00 mmol, 9.64 mL) were stirred at 100 °C for 24 h under nitrogen protection. Then it was extensively washed by *n*-hexane to remove the unreacted 1,6-diisocyanatohexane. The product was dried under vacuum overnight to obtain white powder with the yield of 94%.

Synthesis of PEG-SS-COOH

3,3′-dithiodipropionic acid (15 mmol, 3.16 g), *N,N′*-dicyclohexylcarbodiimide (8.7 mmol, 1.8 g), 4-dimethylaminopyridine (1.00 mmol, 0.12 g) and poly(ethylene glycol) (M_n = 750 g mol^{-1}) (5.00 mmol, 3.75 g) were dissolved in100 mL of THF. After stirring at room temperature for 24 h, the solvent was removed under reduced pressure and the crude product was redissolved in DCM. The cloudy solution was filtrated and washed with 200 mL of brine containing 10 mL of 37% concentration HCl three times. The collected organic layer was concentrated and precipitated in ether/*n*-hexane (v/v = 1/1) for several times to obtain the pure product with the yield of 63.2%.

Synthesis of POSS-P_6-U_2

POSS-(NH_3Cl)$_8$ (0.60 mmol, 0.94 g), UPy-NCO (1.2 mmol, 0.35 g) and triethylamine (1.2 mmol, 0.17 mL) were dissolved in 50 mL of dry dimethyl sulfoxide. After stirring at 60 °C for 6 h under nitrogen protection, white precipitates were collected via precipitation by washing

with DCM for twice to obtain the intermediate product of POSS-$(NH_3Cl)_6$-U_2 with yield of 84.4%. ^{1}H NMR (400 MHz, DMSO-d_6, δ): 16.61 (s, 1H), 9.80 (s, 1H), 7.99 (s, 3H), 7.59 (d, 1H), 6.03 (s, 1H), 5.76 (s, 1H), 3.13 (t, 2H), 2.97 (d, 2H), 2.76 (s, 2H), 2.61 (m, 2H), 2.10 (s, 3H), 1.27 (t, 2H), 1.01 (s, 2H).

POSS-$(NH_3Cl)_6$-U_2 (0.20 mmol, 0.46 g), 1-ethyl-3-(3-dimethylaminopropyl)carbodiimide hydrochloride (4.8 mmol, 0.92 g), *N*-hydroxysuccinimide (4.8 mmol, 0.54 g) and were dissolved in 20 mL of dry dimethyl sulfoxide. Then a solution of PEG-SS-COOH (2.4 mmol, 2.23 g) in 10 mL dry dimethyl sulfoxide and triethylamine (6 mmol, 0.84 mL) were slowly injected into the flask, respectively. After stirring at room temperature for 3 d, it was lyophilized to remove solvent and then purified by dialysis (MWCO 3500) to obtain the pure product with the yield of 88.5%.

Synthesis of POSS-(SS-PEG)$_8$

POSS-(SS-PEG)$_8$ was synthesized according to our previous work [1]. Briefly, PEG-SH (10.2 g, 13.3 mmol) and 2, 2'-dithiodipyridine (8.8 g, 50 mmol) were dissolved in a stirred methanol solution under an argon atmosphere to minimize free thiol oxidation. After stirring for 3 days, the product was precipitated in cold ether for 3 times to give a white powder, PEG-SS-2TP (yield: 80%). ^{1}H NMR ($CDCl_3$, 400 MHz, ppm): δ 2.99 (t, 2H), 3.37 (s, 3H), 3.82 (t, 2H), 7.08-8.45 (m, 4H). ^{13}C NMR ($CDCl_3$, 400 MHz, ppm): δ 38.3, 58.5, 69.6, 70.5, 71.6, 71.8, 120.6, 121.9, 129.1, 149.2 159.6.

Then, POSS-(SH)$_8$ (0.89 g, 0. 88 mmol) was dissolved in 50 mL of chloroform and added dropwise to a stirred solution of PEG-SS-2TP (9.31 g, 10.6 mmol). The reaction was kept under nitrogen atmosphere to minimize free thiol oxidation. The reaction mixture was stirred for 6 days and concentrated to yield a viscous liquid. The product of POSS-(SS-PEG)$_8$ (yield: 68%) was purified by ultrafiltration (MWCO 2000) and collected after freeze-drying. ^{1}H NMR ($CDCl_3$, 400 MHz, ppm): δ 0.73 (t, 2H), 1.76 (m, 2H), 2.41 (t, 2H), 2.83 (t, 2H), 3.37 (s,

3H). ^{13}C NMR ($CDCl_3$, 100 MHz, ppm): δ 10.7, 22.0, 37.8, 41.3, 58.8, 68.6, 69.6, 69.8, 70.5, 71.8.

Preparation of cross-linked hydrogels

50 mg of POSS-P_6-U_2 or POSS-SS-$(PEG)_8$ was dissolved in 445 μL of deionized water with the addition of 5 mg of cysteamine hydrochloride. Then 5 μL of 5 M NaOH was mixed to trigger the reaction. After ultrasonic processing and standing about 1 min, the solution slowly became milky and turned into the loose hydrogel. The hydrogel further shrank into the compact hydrogel if aging longer.

Characterization

^1H NMR spectra were measured on a Bruker DRX-400 spectrometer using $CDCl_3$ and DMSO-d_6 as solvents. Scanning electron microscopy (SEM) images were obtained at acceleration voltage of 5 kV on a JSM-6700F microscope (JEOL, Japan). Rheological measurements were carried out on a Thermo Haake Rheometer equipped with cone-parallel plate geometry (35 mm of diameter) at a gap of 0.5 mm. The sample was measured at 25 $^{\circ}C$ with a constant strain of 0.05% in frequency range of 100-0.1 rad s^{-1}. Fourier transform infrared (FTIR) was taken on a Bruker TENSOR-27 spectrometer in the frequency range 4000-400 cm^{-1}.

Assessment of PDLSCs viability, proliferation and differentiation

The human PDLSCs (hPDLSCs) were acquired form healthy human teeth from orthodontic patients of 10-25 years old without any history of periodontitis. The protocol of hPDLSCs isolation and cultivation was followed according to a previous publication [2]. The hPDLSCs were used for all experiments at Passage 3. All the researches were approved by the Ethics Committee of Peking University (PKUSSIRB-201311103).

Cell Counting Kit-8 (CK04-100T, Dojindo, Japan) was applied to examine the cell viability under manufacturer’s protocol. hPDLSCs were seeded in 24-well plates at a density of 4.0 ×

10^4 mL^{-1}, with the hybrid hydrogels on the bottom of the plates. After 1, 3, and 5 days incubation, 400 uL of culture medium containing 40 uL of CCK-8 solution was added to incubate hPDLSCs for 2 h. The cell viability was measured in Bio-Rad microplate reader at 450 nm wavelength.

For the Live/Dead staining, hPDLSCs were incubated on the hybrid hydrogels for 2 days, the Live/Dead working solution (L3224, Invitrogen, USA) was added according to the manufacturer's instruction. Olympus IX53 fluorescence microscope was used to observe.

The 5-Bromo-2-deoxyuridine (BrdU) assay was used to detect the proliferations of hPDLSCs. After seeding cells on the hybrid hydrogels for 2 days, the BrdU labeling reagent (Thermal Fisher Scientific, USA) was mixed with the culture medium and cells were cultured at 37°C for 12h. Then cells were treated overnight with BrdU antibody (1:200 diluted, Invitrogen, USA). Next, the cells were stained by Alexafluoro 568 conjugated secondary antibody for 1 h at room temperature. Finally, the cells were treated with Vectasheild mounting medium containing DAPI (Solarbio). Zeiss Axio Observer Z1 was used to count percentage of BrdU-positive cells among all the cells.

To evaluate the microtopography of cells with these hydrogel groups substrata, hPDLSCs (1.0×10^4 cells) were seeded and cultivated on the hybrid hydrogels. The samples were fixed with 4% (W/V) paraformaldehyde (PFA); next, every sample was lyophilized by Labconco SPEX 6770 freeze drier, which were then sputtered with Au (99.99%) and observed with a scanning electron microscopy. To assess the microtopography of hPDLSCs influenced by different hydrogels, branching analysis of cells was evaluated based on outline of cells. The primary branching point was defined as the projections descending from the base of the soma (greater than 5 μm); the secondary branching point was determined as the projections from the primary branching (greater than 5 μm); and the tertiary branching points was defined as the projections from the secondary branching (greater than 5 μm) [3].

For ALP staining and ARS staining, hPDLSCs were incubated with osteogenic inducing medium (0.01 μM dexamethasone, 1.8mM KH_2PO_4, 10 mM β-glycerol phosphate, 0.1mM ascorbic acid-2-phosphate in regular growth media), and the hydrogel degradation was added into the medium concurrently, reaching a final concentration of 0.03 wt.%. Alkaline phosphatase (ALP) staining was conducted after 7 days of induction. The cells were rinsed with phosphate buffer saline (PBS) 3 times before fixed in 4% PFA for 20 min at room temperature. Then, washed with PBS 3 times, the cells were incubated in NBT/BCIP solution for 30 min at room temperature, and excessive stain was removed with distilled water. Alizarin red S staining was performed after induction for 14 days. Briefly, after being washed 3 times with PBS, the cells were fixed in 4% PFA for 20min, rinsed 3 times with PBS And then stained with 1% Alizarin red S solution. Excessive dye was removed. Image J (ver. 1.8.0; NIH, USA) were used to record the stained areas

Immunofluorescence assays

For immunofluorescence staining of cultured cells, the hPDLSCs (4×10^4 cells) were seeded and cultivated on the hybrid hydrogels above the chamber slides for 48 h. After fixation with 4% PFA, the cells were permeabilized with 0.01% Triton X-100 (Invitrogen, USA) and blocked with 3% PBS diluted Albumin Bovine V (BSA) for 45 min at room temperature. After rinsed with PBS, the slides were treated with following primary antibodies in 5 wt.% BSA in PBS: anti-vinculin antibody overnight, followed with thorough rinsing. Next, the samples were incubated with the following secondary antibodies for 45 min in the dark: goat anti-rabbit IgG/RBITC, goat anti-mouse IgG/RBITC, and goat anti-mouse IgG/FITC. FITC-Phalloidin was used for cytoskeletal staining. Finally, the cells were mounted by mounting medium containing 4′,6-diamidino-2-phenylindole (DAPI, Solarbio, China). The images were observed and constructed using the Zeiss laser scanning microscope LSM 510.

For immunofluorescence staining of tissue sections, the tissues were fixed in 4% PFA after harvested. And the rat cranial bone tissues were decalcified with ethylenediaminetetraacetic acid (EDTA, pH 7.2). Then both the bone and the skin tissues were embedded in paraffin. The paraffin-embedded sections were blocked with serum, and incubated with primary antibodies overnight at 4 °C. Then the sections were treated with FITC or Rhodamin-conjugated secondary antibodies and mounted by mounting medium containing DAPI (DAPI, Solarbio, China). The images were observed and constructed using Olympus BX53 microscope (Japan).

Quantitative polymerase chain reaction (qPCR)

The total RNA from hPDLSCs encapsulated in hydrogels was isolated by TRIzol reagent (Invitrogen), and reverse-transcribed to cDNA by a PCR thermal cycler (Takara, Japan). Quantitative PCR was performed on ABI Prism 7500 Real-Time PCR System (Applied Biosystem, USA). Each well was loaded with 20 μL PCR mixture, including 10 μL RNase-free water, 8 μL FastStart Universal SYBR Green Master Mix (Roche, Japan), 1 μL primer and1 μL template cDNA. The cycling parameters of PCR amplification were as follows: 15 min at 95 °C, 40 cycles (15 s each cycle) at 95 °C, and then 1 h at 60 °C. The expression levels of gene markers were normalized by GAPDH. The primers sequences were indicated in **Table S1.**

Western blot analysis and the co-immunoprecipitation assay

hPDLSCs encapsulated in hydrogels were lysed using radioimmunoprecipitation assay (RIPA) buffer (Thermo Fisher Scientific), containing a Halt™ protease, phosphatase inhibitor cocktail and a ethylene diamine tetraacetic acid (EDTA) solution. After centrifugation for 30 min (12,000 rpm, 4 °C), the supernatants were gathered. Protein concentration was measuredusing a bicinchoninic acid (BCA) protein assay kit (Beyotime, China). Then, the samples were mixed with 4× sodium dodecyl sulfate (SDS) loading buffer (Solarbio, China) at a 1:3 ratio and heated for 5 min at 90 °C for protein degradation. Twenty micrograms

applied proteins were separated on 4-12% NuPAGE gel (Solarbio, China) and then transferred to polyvinylidene fluoride (PVDF) membranes. The membranes were blocked with 5% BSA (diluted with TBST) for 1 h, incubated in primary antibodies overnight according to manufacturers' instructions, and incubated with HRP-conjugated secondary antibodies for 1 h at room temperature. The SuperSignal® West Pico Chemiluminescent Substrate (ThermoFisher, USA) and BioMax film (Kodak, USA) were applied for the detection of immunoractive proteins. β-actin was used as the internal control.

For the co-immunoprecipitation assay, the immunoprecipitations were conducted with anti-TET2, anti-HDAC1 or normal rabbit IgG at 4 °C overnight, after trimming the protein concentration. And then, the immunoprecipitates were incubated with ProteinIso® Protein A/G Resin for 2h. After thoroughly wash with lysis buffer, the immune products were resolved by SDS-PAGE (Solarbio, China) and evaluated by western blot.

Evaluation of bone regeneration in rat cranial bone defect model

All the animal experiments were approved by Peking University Biomedical Ethics Committee (LA20190074). Healthy SD rats (aged 6-8 weeks, female) employed for calvaria bone defects and transplantation treatments were purchased from The VitalRiver. The rats were randomly divided into 5 groups: (1) Control; (2) PEG; (3) POSS-P_8-0.5 h; (4) POSS-P_6-U_2 0.5 h; (5) POSS-P_6-U_2 12 h. There were 8 rats in each group. The group without any additives was used as control. The hydrogels were cut into a diameter of 5 mm and height of 1-2 mm before surgery and incubated with 2.0×10^5 mL^{-1} hPDLSCs for 48 h, then the PDLSCs-hydrogel complexes were washed by PBS before implantation [4]. The 5 mm calvaria bone defects were fabricated by stainless-steel trephine on both sides of the skull. The defect areas were filled by pretreated hydrogel with hPDLSCs. At 8 weeks after operation, The rats were sacrificed with 1% sodium pentobarbital solution. The calvaria bones were isolated and fixed in 4% neutralbuffered formaldehyde and then subjected to

microcomputed tomography (μCT, SkyScan 1174) scanning to detect bone formation. The index including bone mineral density (BMD) and bone volume to total volume (BV/TV) were also quantitatively assessed. Serial sections (5 μm thick) were stained through hematoxylin and eosin (H&E), Masson staining and immunofluorescence staining to assess regenerated bone and inflammation status.

Assessment of biocompatibility and degradability of subepidermal implanted hydrogels

Healthy C57BL/6J mice (aged 8 weeks, female) were purchased from The VitalRiver to evaluate the toxicity of the hybrid hydrogels. The mice were randomly assigned to 5 groups: (1) Control; (2) PEG; (3) POSS-P_8-0.5 h; (4) POSS-P_6-U_2 0.5 h; (5) POSS-P_6-U_2 12 h. There were 8 mice in each group. The group without any additives was used as control. After 2 and 8 weeks of subcutaneous implantation of the hydrogels on the dorsum of the mice, the skin and implants were harvested and fixed for 2 days. Then the samples were dehydrated and embedded in PMMA, and sections (3 μm) were created and treated using H&E staining and immunofluorescence staining for toxicity and inflammation evaluation.

Knockdown assays

To knockdown Tet2 expression in hPDLSCs, the cells were treated with Tet2 siRNA or the vehicle siRNA control with lipofectamine reagent (13778158, thermo, USA) following the manufacturer’s instructions. To inhibit HDAC1 expression, the hPDLSCs were treated with Entinostat (MS275) or DMSO after cultured under the reduced serum medium (Opti-MEM, Gibco, USA).

Statistical Analysis

For the cell viability analysis, CCK-8 assay, BrdU-positive cell percentage analysis, quantitative analysis of Vinculin-positive area percentage, analysis of number of branching points, qPCR assay, and evaluation of differentiation, the data were presented as mean ± standard deviation. Statistical comparisons between two groups were assessed using Student’s

t-tests, and one-way ANOVA was carried out when more than two groups were compared by using Graphpad Prism 8 software. P values < 0.05 were designated as statistically significant.

Table S1. Sequences of primers used for qPCR analysis.

Gene	Forward primer (5′-3′)	Reverse primer (5′-3′)
GAPDH [a)]	GGAGCGAGATCCCTCCAAAAT	GGCTGTTGTCATACTTCTCATGG
OCN [b)]	CACTCCTCGCCCTATTGGC	CCCTCCTGCTTGGACACAAAG
ALP [c)]	AACATCAGGGACATTGACGTG	GTATCTCGGTTTGAAGCTCTTCC
RUNX2 [d)]	TGGTTACTGTCATGGCGGGTA	TCTCAGATCGTTGAACCTTGCTA

[a)]GAPDH, glyceraldehyde-3-phosphate dehydrogenase; [b)]OCN, osteocalcin; [c)]ALP, alkaline phosphatase; [d)]RUNX2, runt-related transcription factor 2.

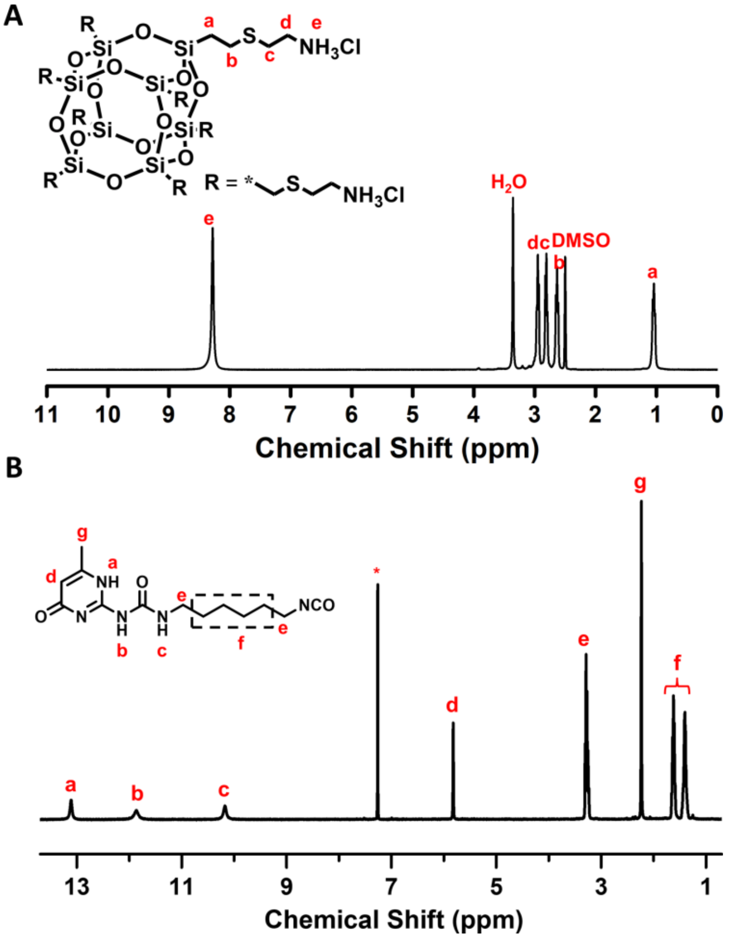

Figure S1. (A)^{1}H NMR spectrum of POSS-$(NH_3Cl)_8$ at DMSO-d_6 and (B) UPy-NCO at $CDCl_3$.

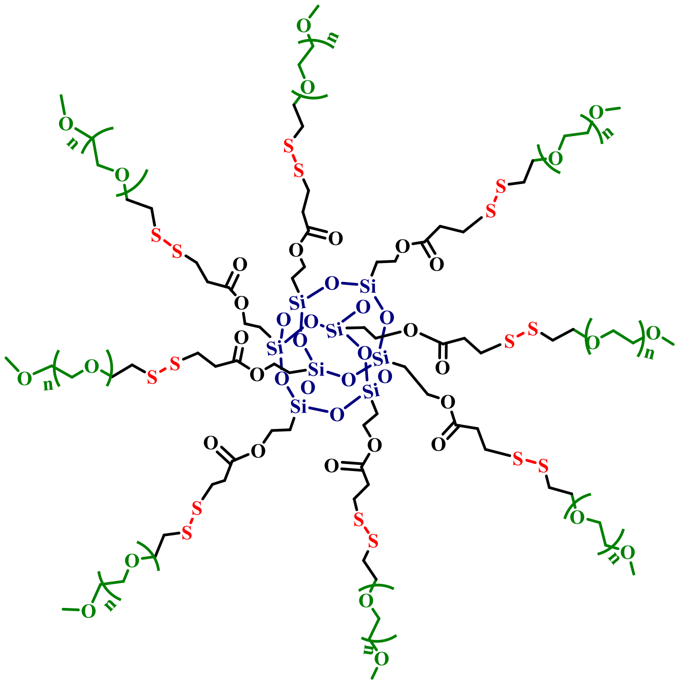

Figure S2. The structure of POSS-(SS-PEG)$_8$ molecule.

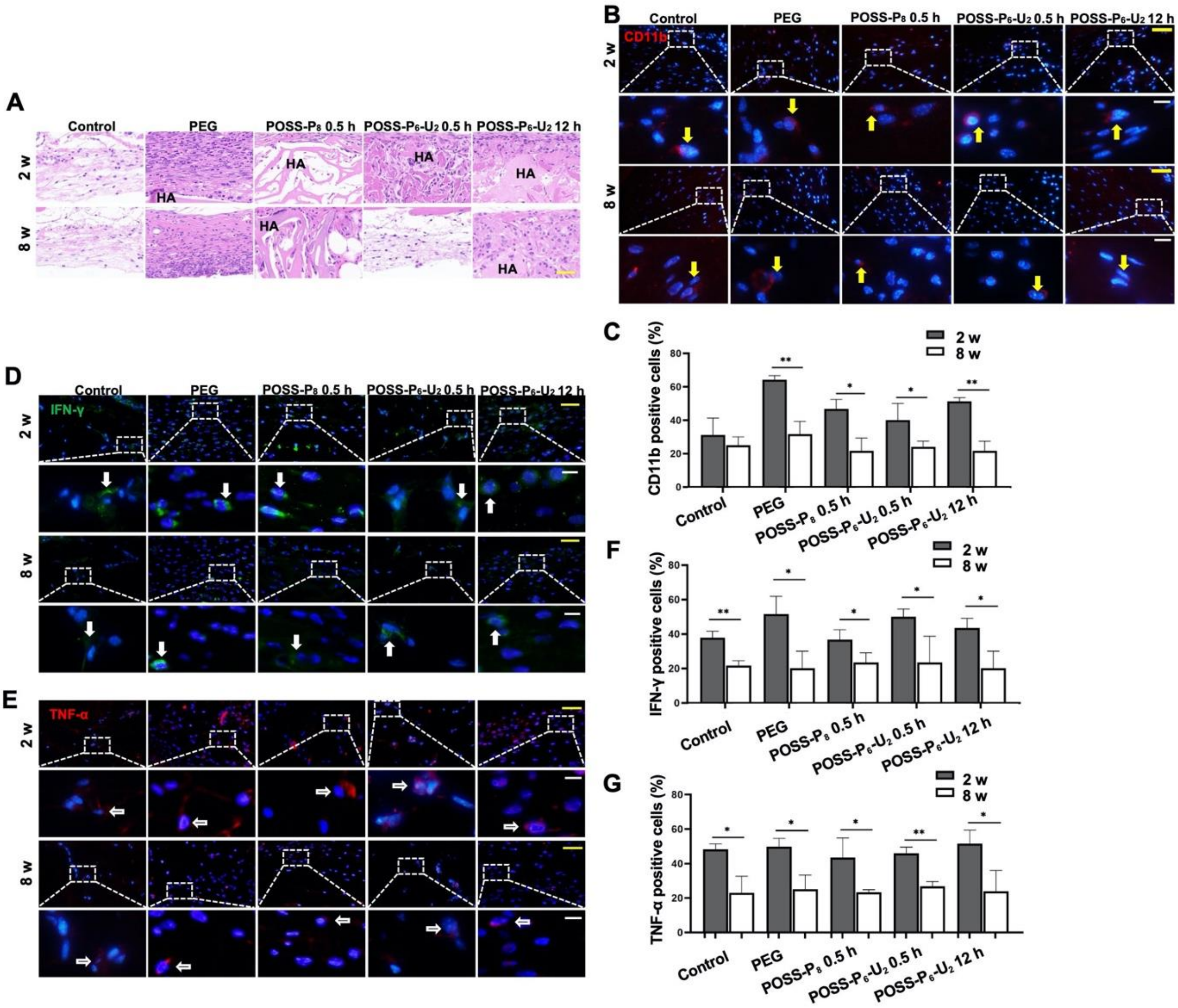

Figure S3. Histological analysis of the subcutaneous hydrogel implant areas. (A) The H&E staining of the hydrogel implant areas in C57BL/6J mice 2 and 8 weeks after implantation. HA represents the residual hydrogels. (B) Immunofluorescence staining and semi-quantitative analysis (C) of CD11b (yellow arrows), (D) IFN-γ (solid arrows) and (E) TNF-α (non-solid arrows) in the hydrogel implant areas in C57BL/6J mice 2 and 8 weeks after implantation. Semi-quantitative analysis of (F) IFN-γ and (G) TNF-α positive cells is also included. Scale bar: 50 μm (yellow), 20 μm (white). (n=3 per group; *$p < 0.05$, **$p<0.01$)

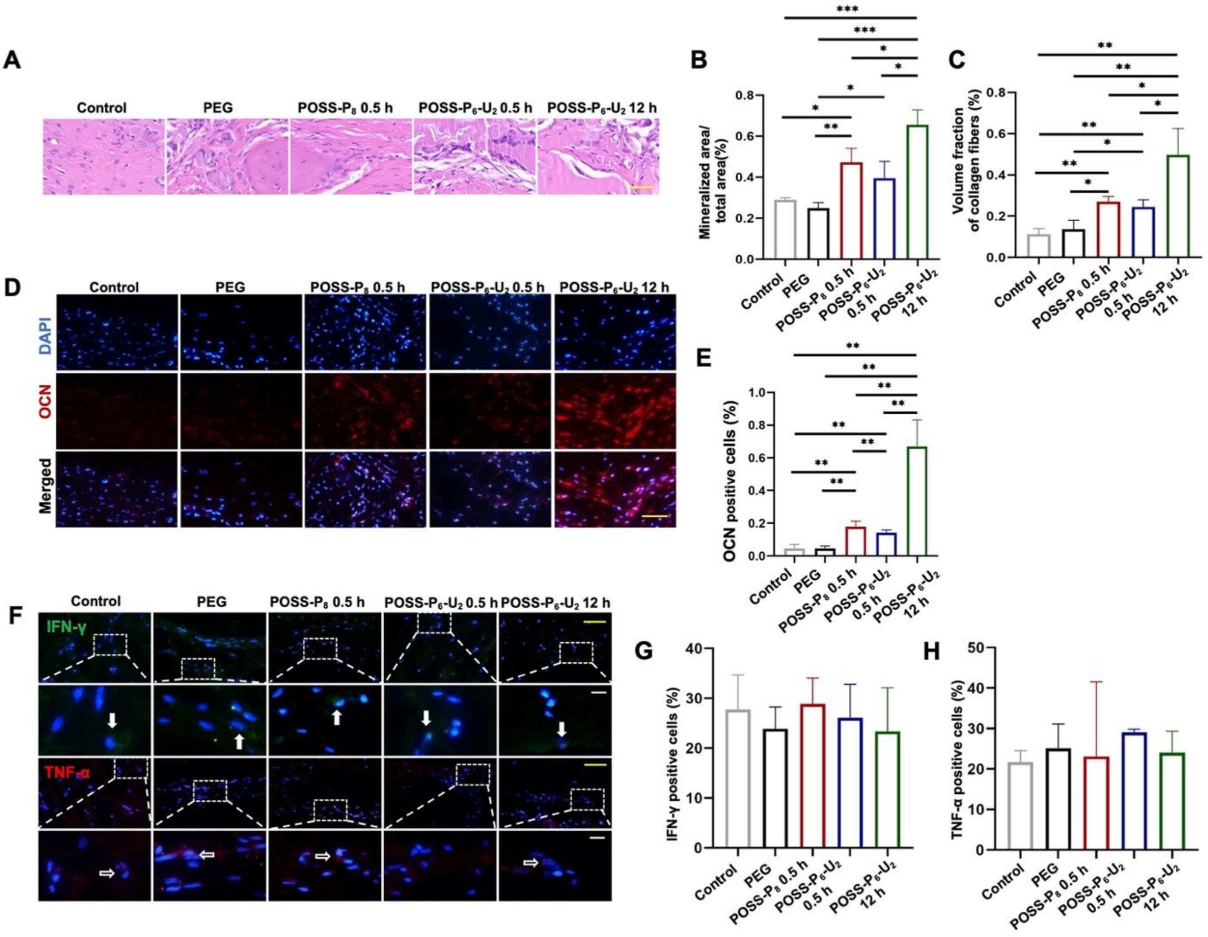

Figure S4. Inflammatory and osteogenesis analysis of the calvarial defect areas. (A) The H&E staining of the calvarial defect areas 8 weeks after implantation. (B) Semi-quantitative analysis of H&E and (C) Masson's trichrome staining around the calvarial defect areas in SD rats 8 weeks after surgery. (D) Immunofluorescence staining of OCN around the calvarial defect areas in SD rats 8 weeks after surgery. (E) Semi-quantitative analysis of OCN positive cells is also included. (F) Immunofluorescence staining of IFN-γ (solid arrows) and TNF-α (non-solid arrows) around the calvarial defect areas in SD rats 8 weeks after surgery. Semi-quantitative analysis of (G) IFN-γ and (H) TNF-α positive cells is also included. Scale bar:50 μm (yellow), 10 μm (white). (n=3 per group; $^*p < 0.05$, $^{**}p < 0.01$, $^{***}p<0.001$)

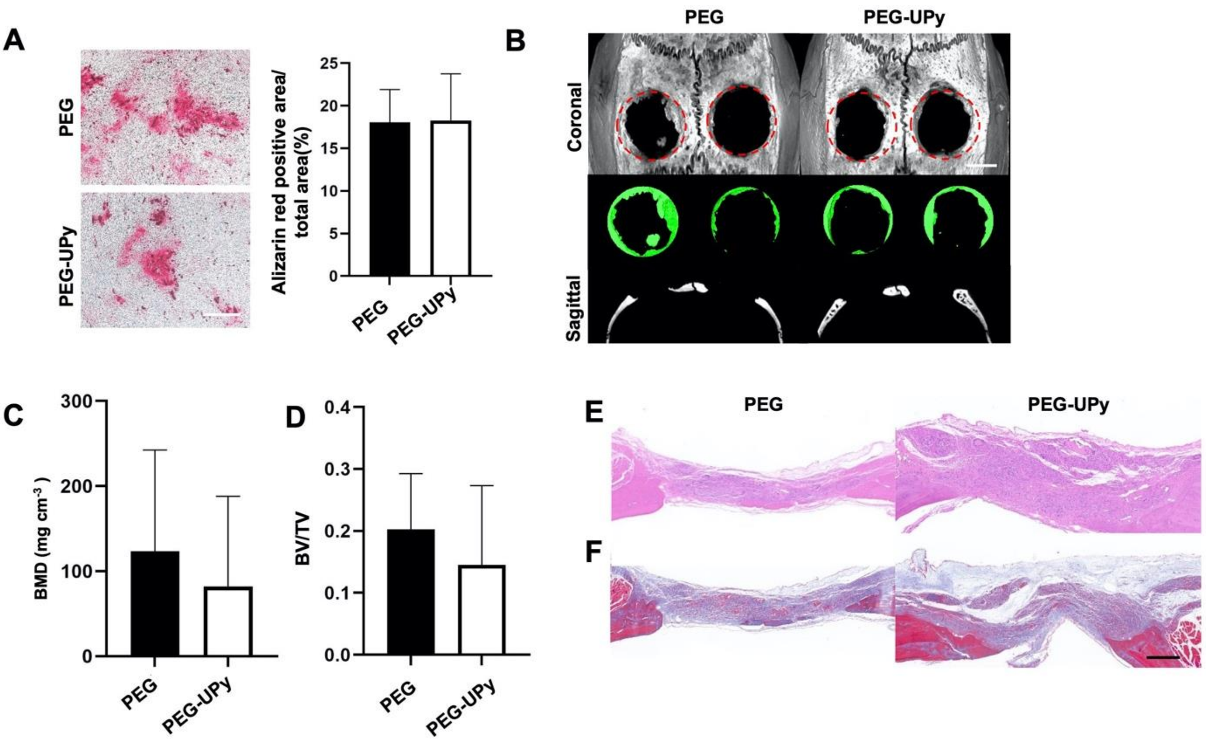

Figure S5. The effect of UPy moiety on osteogenic differentiation of PDLSCs *in vitro* and *in vivo*. (A) ARS staining and the positive area percentages revealing the osteogenic differentiation of MSCs in PEG and PEG-UPy groups. Scale bar: 500 μm. (B) 3D reconstruction of μCT images of new bone formation in rat cranium at 8 weeks after MSC/hydrogels implantation. Scale bar: 5 mm. Quantitative analysis of the bone mineral density (BMD) (C) and bone volume/tissue volume (BV/TV) (D) of regenerated bone tissue. HE staining (E) and Masson's trichrome staining (F) at 8 weeks after implantation. Scale bar, 500 μm. (n=3 per group; *$p < 0.05$)

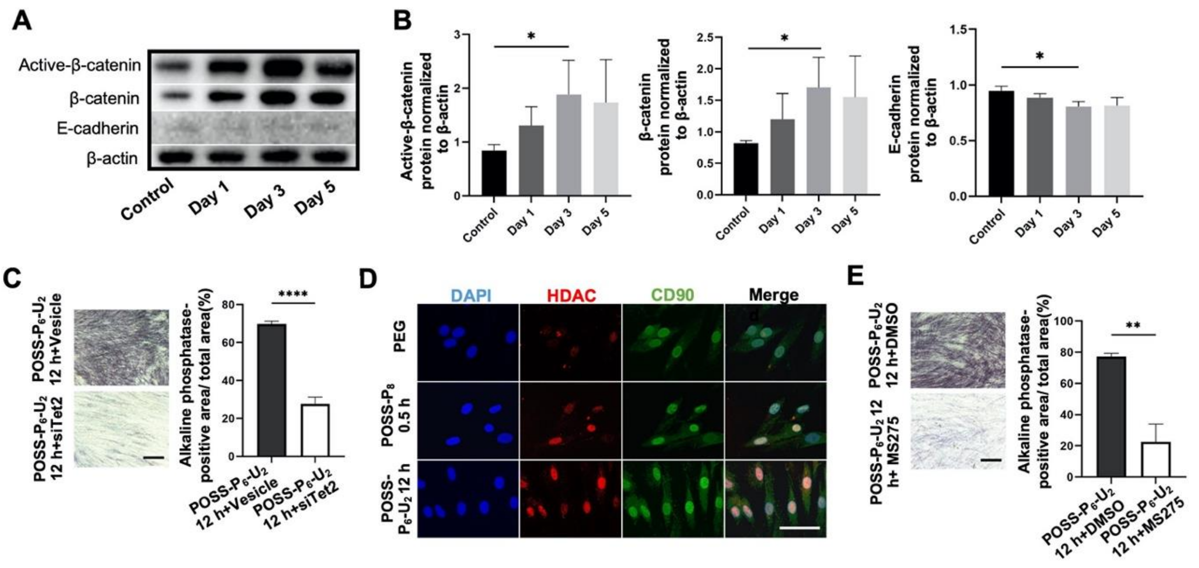

Figure S6. Supplementary demonstration on osteogenic mechanism of POSS-P_6-U_2 12 h hydrogels. (A) Western blotting analysis showing the levels of Active-β-catenin, β-catenin, and E-cadherin in PDLSCs on day 1, 3, 5 after cultured on POSS-P_6-U_2 12 h hydrogels. (B) Semi-quantification analysis of the western blot expression levels. (C) ALP staining and the positive area percentages revealing the osteogenic differentiation of PDLSCs after the knockdown of Tet2. Scale bar: 500 μm. (D) Immunofluorescence analysis showing the expression of HDAC1 in PDLSCs on the POSS-P_6-U_2-12 h, POSS-P_8-0.5 h, and PEG hydrogels. Scale bar: 50 μm. (E) ALP staining and the positive area percentages showing the osteogenic differentiation of PDLSCs after the knockdown of HDAC1. Scale bar: 500 μm. (n=3 per group; *$p < 0.05$, **$p < 0.01$.)

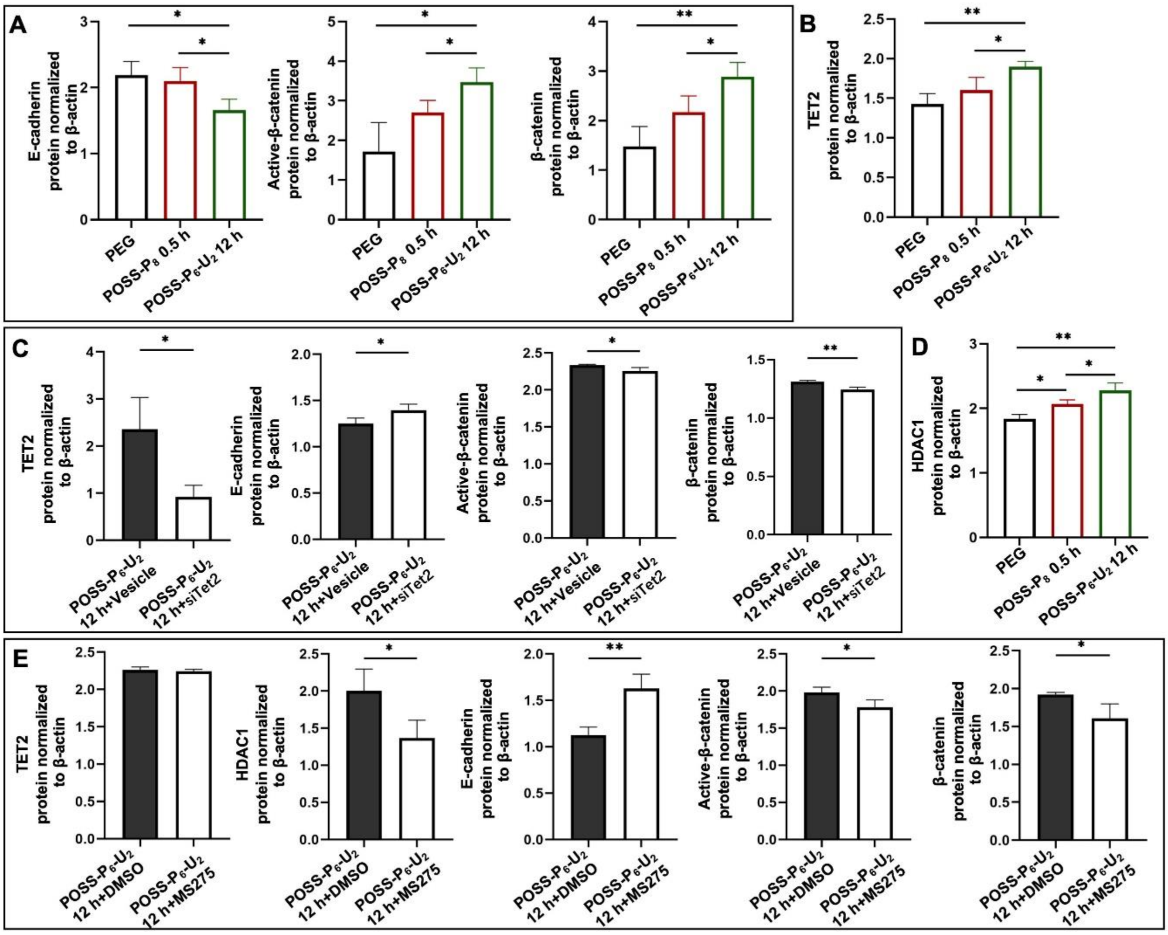

Figure S7. Semi-quantification analysis of the western blot expression levels in Figure 6. (n=3 per group; *$p < 0.05$, **$p < 0.01$)

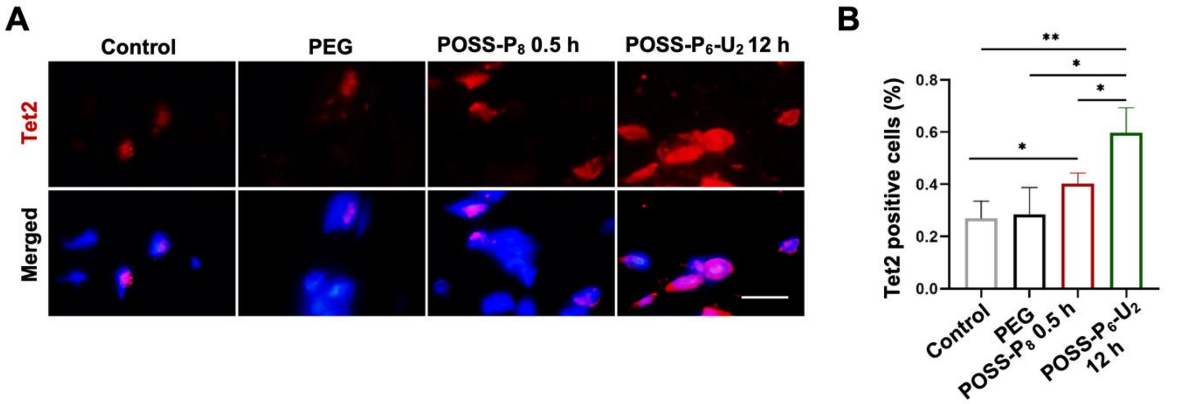

Figure S8. (A) Immunofluorescence staining of Tet2 around the implantation site in calvarial defect areas in SD rats eight weeks after surgery. (B) Semiquantitative analysis of Tet2 positive cells is also included. Scale bar: 10 μm (white). (n=3 per group; *$p < 0.05$. **$p < 0.01$)

[1] X. Wang, P.Y. Gao, Y.Y. Yang, H.X. Guo, D. C. Wu, *Nat. Commun.* **2018**, 9, 2772.

[2] B.M. Seo, M. Miura, S. Gronthos, P.M. Bartold, S. Batouli, J. Brahim, M. Young, P.G. Robey, C.Y. Wang, S. Shi, *Lancet* **2004**, 364, 149.

[3] a) T.T.Yu, F.Z. Cui, Q.Y. Meng, J. Wang, D.C. Wu, J. Zhang, X.X. Kou, R.L. Yang, Y. Liu, Y.S. Zhang, F. Yang, Y.H. Zhou, *ACS Biomater. Sci. Eng*. **2017**. 3, 1119; b) Y. Fu, S. Liu, S.J. Cui, X.X. Kou, X.D. Wang, X.M. Liu, Y. Sun, G.N. Wang, Y. Liu, Y.H. Zhou, *ACS Appl. Mater. Interfaces*. **2016**. 8, 15958.

[4] Y.F. Zhang, X.Y. Dou, L.Y. Zhang, H.F. Wang, T. Zhang, R.S. Bai, Q.N. Sun, X. Wang, T.T. Yu, D.C. Wu, B. Han, X. L. Deng, *Bioact. Mater*. **2022**, 11, 130.
